# Pneumatosis Intestinalis: A Case Report and Approach to Management

**DOI:** 10.1155/2011/571387

**Published:** 2011-02-09

**Authors:** Sean Donovan, Joseph Cernigliaro, Nancy Dawson

**Affiliations:** ^1^Department of Medicine, Mayo Clinic, Jacksonville, FL 32224, USA; ^2^Department of Radiology, Mayo Clinic, Jacksonville, FL 32224, USA

## Abstract

Pneumatosis intestinalis (PI), defined as gas within the bowel wall, is an uncommon radiographic sign which can represent a wide spectrum of diseases and a variety of underlying diagnoses. Because its etiology can vary greatly, management of PI ranges from surgical intervention to outpatient observation (see, Greenstein et al. (2007), Morris et al. (2008), and Peter et al. (2003)). Since PI is infrequently encountered, clinicians may be unfamiliar with its diagnosis and management; this unfamiliarity, combined with the potential necessity for urgent intervention, may place the clinician confronted with PI in a precarious medical scenario. We present a case of pneumatosis intestinalis in a patient who posed a particularly challenging diagnostic dilemma for the primary team. Furthermore, we explore the differential diagnosis prior to revealing the intervention offered to our patient; our concise yet inclusive differential and thought process for rapid evaluation may be of benefit to clinicians presented with similar clinical scenarios.

## 1. Introduction

Pneumatosis intestinalis (PI) is defined simply as the radiographic finding of gas within the bowel wall. PI is an uncommon entity which has recently come to increased clinical attention due to improved radiographic identification; others have cited the radiographic incidence of PI to be present in 0.37% of patients who have abdominal CT scans [1]. Despite this, the authors' review of the literature was dominated by case reports and small case series with only a few large retrospective studies identified [1–4]. Currently, there is no consensus on the appropriate management of PI. Others have attempted to create algorithms for the management of PI, and while helpful, these tedious algorithms may be difficult to apply clinically when the patient needs rapid evaluation [4]. 

Since PI can represent a wide range of pathology, it is in itself not diagnostic of any certain condition. This finding can represent pathologies which range from life-threatening to benign, and, for this reason, management of PI can range from urgent surgical intervention to outpatient observation. Delineating between these etiologies may be difficult but is important to establish with overall mortality estimated to be between 20% and 25% [1, 2, 4]. The variety of presentations of PI highlight the fact that clinicians should interpret radiographic findings in concert with the current clinical scenario in order to ensure a correct diagnosis and to guide one toward a suitable management. The authors emphasize that early recognition of the overall clinical picture is perhaps most important for decision making, with focus placed on key clinical features to efficiently distinguish between life-threatening and nonurgent causes of PI.

## 2. Case Report

An 84-year-old white female with a history of atrial fibrillation, remote history of colorectal cancer treated with a partial colectomy with colostomy and postoperative radiation, and remote history of numerous small bowel obstructions necessitating resection of the terminal ileum with mild residual short gut syndrome presented with a 3-week history of diarrhea and cramping abdominal pain. The diarrhea consisted of six to eight episodes of voluminous, watery, loose, and brown stool without hematochezia or melena. Pain was mild, cramping, diffuse, and worse with food. There was no tenesmus, and the patient denied nausea or vomiting. No fever, chills, myalgias, or systemic signs of illness were present. Past surgical history was notable for remote hip arthroplasty and lower lumbar fusion. Review of systems was otherwise unremarkable.

Physical examination revealed a temperature of 36.8°C, blood pressure of 132/68 mm Hg, pulse of 72 beats per minute, 18 respirations per minute, and oxygen saturation of 99% on ambient air. The patient was a nontoxic female in no acute distress. Cardiovascular exam showed an irregularly irregular rhythm with regular rate. Pulmonary examination demonstrated lungs which were clear to auscultation; there was no wheezing, rales, or rhonchi. Her abdominal exam revealed a soft abdomen with no significant tenderness to light palpation; however there was diffuse tenderness to deep palpation. Brown, loose stool, and gas was found in the patient's colostomy bag. There was no rebound or guarding, and bowel sounds were normal. Initial laboratory investigations were unremarkable, and pertinent negative labs included a normal white blood cell count and nonelevated lactate (Table 1).

**Figure 1:**
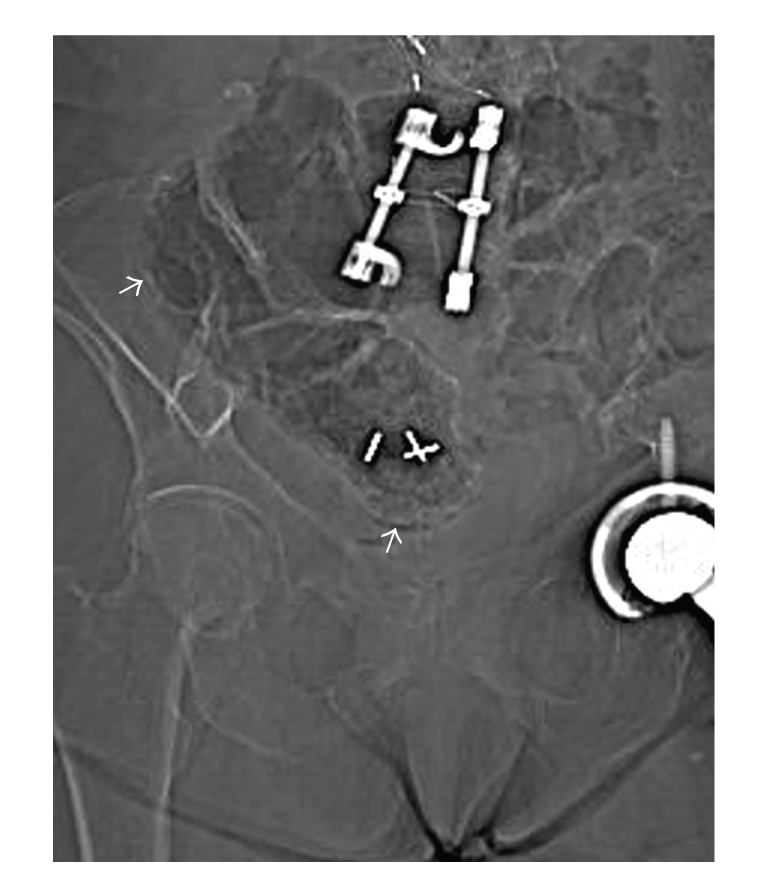
Scout film from abdominal and pelvic CT scan shows crecentic lucencies in the walls of the stool-filled cecum and ascending colon, compatible with pneumatosis.

**Figure 2:**
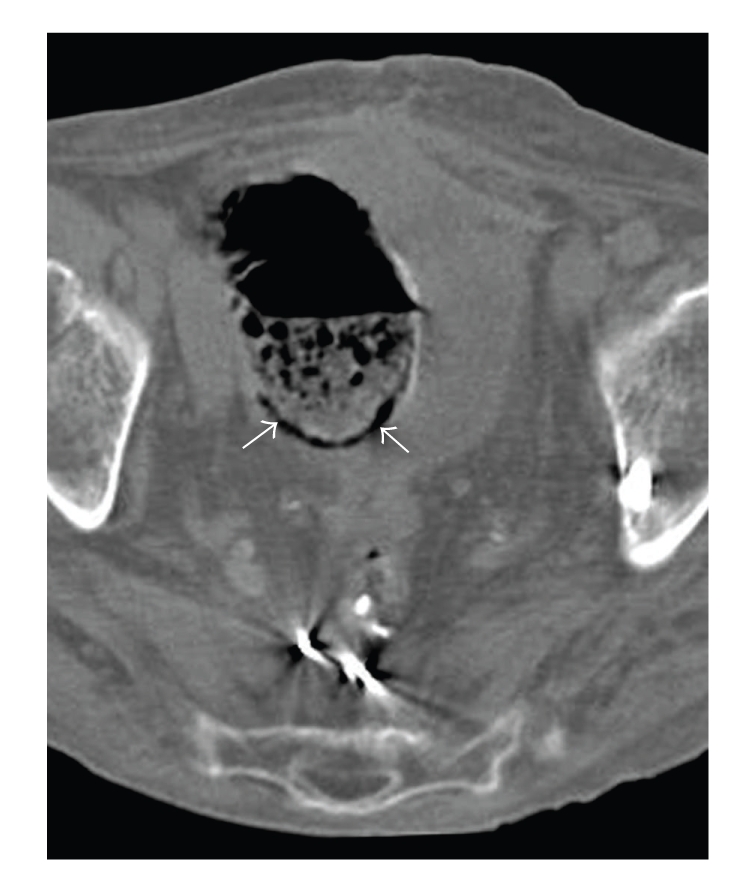
Axial noncontrast image of the pelvis shows a curvilinear collection of gas in the dependent wall of the large bowel (arrows). The dependent location of the gas helps distinguish pneumatosis from intraluminal air.

The CT scanogram demonstrated left lower quadrant bowel gas-filled loops, with one loop demonstrating an additional crescentic gas lucency (Figure 1). The bowel gas pattern was nonobstructive; there was no free air within the abdomen. The corresponding CT scan of the abdomen without IV contrast was performed demonstrating air within the wall of the bowel wall, especially the pelvic cecum and the patient's remaining rectum (Figure 2). There was no bowel wall thickening.

## 3. Discussion

Based on the radiographic finding of air within the bowel wall seen on CT, the patient was diagnosed with pneumatosis intestinalis (PI). The pathogenesis of pneumatosis is currently thought to be the result of many contributing factors [5]. However, the development of PI can be broken down into two main components. The first component is the mechanical aspect of gas traversing the mural portion of the bowel. This can be precipitated by microbreaks in the mucosa, such as those caused by inflammation or necrosis; it can also be the result of direct gas diffusion across an intact mucosal membrane, as can occur in instances of increased transabdominal pressure [6, 7]. The second aspect is the origin of the gas; while some amount of intramural gas is normally present in the human bowel, bacterial overgrowth and invasion of the bowel wall can result in excess gas production favoring the formation of PI [8]. 

The composition of the gas which results in PI has been well studied and gives insight into PI's formation and etiology. The gas-filled colonic cysts in patients with chronic pneumatosis intestinalis have previously been identified as containing nitrogen, hydrogen, and carbon dioxide, but interestingly not the typical colonic gas methane [9]. Florin has hypothesized that idiopathic PI occurs through the phenomenon of “counterperfusion supersaturation,” which discusses the exchange of hydrogen and nitrogen between the blood delivered to the colon and the colonic lumen itself [10]. Essentially, this is comparable to decompression sickness in deep sea divers, except with normal atmospheric pressure. The “supersaturation” component of this concept is hinged upon Henry's Law, which states that the solubility of a gas dissolved in a liquid is directly proportional to the pressure of that same gas above the liquid. In decompression sickness, the initial increase in pressure allows for a gas (nitrogen) to dissolve into a solution (the blood) per Henry's Law, and the subsequent rapid decrease in pressure provides the scenario whereby the gas previously dissolved in solution can precipitate out—forming bubbles. Therefore, the formation of gas pockets and bubbles occurs only if there is some initial degree of gas supersaturation into solution. However, in humans under conditions of normal atmospheric pressure, supersaturation of gas into the colonic lumen is unlikely to occur as a result of simple increases in colonic lumenal pressure, since passing flatus would alleviate this increase in pressure; supersaturation must occur in the absence of increases of pressure as it occurs in decompression sickness.

This is where the “counterperfusion” aspect comes into play and provides a model whereby supersaturation can occur without increases in pressure. In this model, two inert gases attempt to travel across their normal diffusion gradients, but this exchange of gases is limited by two resistances. In the human model, the two inert gases are hydrogen, which is typically found in the lumen of the gut due to production by bacteria, and nitrogen, which is found in the blood as a result of pulmonary gas exchange. It had been identified that many patients with idiopathic PI are H_2_ superproducers [10]. Typically, hydrogen is excreted through flatus or breath, which lowers hydrogen luminal tension and eliminates the possibility of hydrogen approaching the tension of nitrogen in the blood, which creates a steady state for both hydrogen and nitrogen tension. However, in hydrogen superproducers, the increase in hydrogen tension due to colonic bacteria provides a scenario in which the luminal hydrogen tension can approach that of the nitrogen in the blood. As the increase in hydrogen lumen tension occurs, it attempts to travel down its diffusion gradient into the blood; conversely, as the relative tension of lumenal nitrogen decreases, nitrogen from the blood travels into the colonic lumen. However, this process is limited by the resistive index of the colonic wall itself and does not allow for smooth exchange of gas. In this fashion, cyst formation within the colonic wall is favored and thereby explains pneumatosis intestinalis. This “counterperfusion supersaturation” model additionally explains why patients who produce large amounts of methane—a gas which does not have the diffusivity of nitrogen—would not be associated with PI.

Furthermore, other authors have examined the relationship between alkyl halides and pneumatosis intestinalis [7]. Others have attempted to the association which had been previously identified between patients taking choral hydrate, one of the oldest synthetic hypnotic agents, and PI. Chloral hydrate degrades rapidly into its active metabolite trichloroethanol and other metabolites which are classified as alkyl halides; these substances are known to inhibit the hydrogen consumption by methanogens. Therefore, utilization of these substances can increase hydrogen lumenal tension through diminished hydrogen consumption. Furthermore, in in vitro models, exposure to alkyl halides significantly increased the amount of hydrogen produced by anaerobic fecal cultures. This increase was particularly marked in feces derived from patients who produced methane. Similarly, in the in vivo models, hydrogen levels were increased in the rats used for the experiment. In this manner, hydrogen lumenal tension is increased through a variety of mechanisms. The high rate of PI found in patients using alkyl halide precursors, and known mechanism of increased hydrogen lumenal tension, gives more credence to the theory of counterperfusion supersaturation leading to PI and additionally explains the association between alkyl halides and PI.

Prior authors have devised multiple methods to classify the expansive differential diagnosis which encompasses PI. A more inclusive list of conditions associated with PI is discussed elsewhere [5]. From a clinician's view, however, an initial clarification between life-threatening and nonurgent pathologies is of utmost importance (Table 2). Most concerningly, PI may be indicative of necrotic tissue allowing gas to penetrate the submucosa. Mesenteric ischemia from low-flow states or infarction from acute arterial occlusion may be the most concerning of the high risk etiologies and can give rise to this clinical picture [11]. A high index of suspicion warrants an urgent intervention and consideration of surgical consultation for suspected ischemia or infarction. Other urgent pathologies which can be associated with ischemia include intestinal obstruction as well as volvulus and malrotation.

When considering ischemia or infarction, rapid recognition of the overall clinical picture is imperative. There are several key features of the current clinical scenario, patient past medical history, physical exam, and laboratory data, which can heighten suspicion for life-threatening PI (Table 3). An immediate appreciation of low-flow vascular states, such as sepsis, CHF, use of IV pressors, and other causes of hypotension, should be made by the physician. Arrhythmias, which can cause both low-flow states as well as precipitate embolic phenomena, should also be taken into account. The patient's past medical history should be examined for peripheral vascular disease and coronary artery disease as well as risk factors for vascular disease such as hypertension, hyperlipidemia, diabetes, and smoking. Physical examination should focus upon the abdomen, with caution being given to an exam revealing the classic ischemic finding of “pain out of proportion to exam.” Signs of peritonitis, although uncommonly present, may also suggest ischemia [4]. Laboratory data should include a lactate level, with an elevated lactate raising serious concern for ischemia [2]. 

Furthermore, details of the radiographic findings can help define the nature of PI. The finding of additional gas in the vasculature, particularly portal venous gas, can be an ominous sign and correlates to transmural bowel necrosis [2]. Other authors have suggested that so-called crescentic or linear gas collections may indicate bowel infarction and are more often associated with more sinister pathology [12]. This should be contrasted with so-called cystic PI, which represents discrete bubbles of gas attached to one another along the digestive tract wall and is usually considered benign. Small bowel PI is more frequently associated with ischemia than large bowel PI alone [4]. Thus, radiographic details may be used to aid in identifying life-threatening causes of PI.

After the more serious etiologies have been ruled out, nonurgent pathologies should be considered. Mechanical trauma such as recent surgical anastomosis and endoscopy can impair the normal mucosal barrier and may represent the underlying cause [11, 13]. The differential also includes an abundance of infectious etiologies which can cause inflammation and thereby induce microbreaks in the mucosa. Perhaps the most prominent of these is Clostridium Difficile [14]. This infectious differential expands with the immunocompromised host to include such etiologies such as cytomeglovirus, mycobacteria, pneumocystis carinii, and the HIV/AIDS virus itself [15]. Alternatively, autoimmune processes such as the inflammation stemming from Crohn's disease and ulcerative colitis may manifest as PI [16]. Conversely, any localized condition causing increased translumenal pressure such as chronic pseudo-obstruction may cause PI [17]. Furthermore, cases associated with immunosuppression and, rarely graft-versus-host disease have been described [18, 19]. Despite all these possibilities, a large proportion of patients have benign idiopathic disease [4].

The cause of pneumatosis intestinalis is not exclusively limited to GI tract pathology. While debatable, it has been suggested that cystic fibrosis, asthma, and other obstructive bronchopathologies such as COPD can cause PI [20]. This may be due to these entities resulting in chronic cough which increase transabdominal pressure and could thereby cause transmucosal air dissection. 

Interestingly, location of the pneumatosis intestinalis which can be anywhere from the stomach to the rectum may guide the clinician toward the cause. More proximal disease can represent rarer causes of PI such as pyloric stenosis, gastric ulcer, or gastric malignancy while distal lesions may stem from pathology such as appendicitis or diverticulitis.

Taking a “worst case scenario” approach may be of particular benefit towards patients with PI. This tactic will help the clinician in expeditiously ruling out urgent pathology while ultimately leading to a diagnosis. In this particular instance, the clinical context and radiographic findings did not suggest urgent pathology. Nevertheless, a surgical consultation was ordered as the patient had “pain out of proportion to the physical exam” and therefore could have been suffering from subacute bowel infarction. After evaluation by surgery, it was noted that the lactate was normal and urgent surgical exploration was not deemed necessary. With this and other serious pathologies also ruled out, pneumatosis intestinalis secondary to a less emergent cause was considered. While the patient did have a history of COPD, absence of signs and symptoms of COPD exacerbation and history of prior CTs without evidence of PI make COPD an unlikely cause. Further workup revealed a negative stool WBC and Clostridium difficile toxin. With no other plausible cause of PI, the patient's presentation was ultimately thought to represent a mild bacterial infection yielding minimal clues to the definitive diagnosis aside from radiographic data. The patient was treated with metronidazole and her diarrhea resolved without sequelae, further reinforcing the diagnosis.

## 4. Conclusion

It is important to recognize that pneumatosis intestinalis is a clinical sign and is in itself not a diagnosis. Because it represents such a wide spectrum of diseases, rapid evaluation of PI may be difficult. An efficient recognition of the clinical scenario, encompassing the current clinical context, comorbid conditions, physical examination findings, laboratory data, and radiographic details, assists the clinician in reaching the correct diagnosis and offering appropriate treatment.

## Figures and Tables

**Table 1:** Laboratory data.

Variable	Reference range, adults	On admission
Hemoglobin (g/dL)	12.0–15.5	13.6
Hematocrit (%)	34.9–44.5	42.5
White cell count (per mm3)	3500–10500	4400
Platelet count (per mm3)	150,000–450,000	169,000

Sodium (mEq/dL)	135–145	145
Chloride (mEq/dL)	100–108	106
Potassium (mEq/dL)	3.5–5.1	4.7
Bicarbonate (mEq/dL)	22–29	28
BUN (mg/dL)	12–21	26
Creatinine (mg/dL)	0.7–1.2	1.4

Ionized calcium (mg/dL)	4.7–5.4	4.6
Ionized magnesium (mmol/L)	0.50–0.73	0.40

Lactate (mmol/L)	0.9–1.7	0.5

Alkaline phosphatase (units/L)	55–142	92
AST (units/L)	12–31	20
ALT (units/L)	9–29	12
Total bilirubin (mg/dL)	0.1–1.1	0.3
Direct bilirubin (mg/dL)	0.0–0.3	0.1

**Table 2:** Prominent causes of pneumatosis intestinalis.

Nonurgent	Life-threatening
Traumatic	(i) Ischemia
(i) Surgical anastamosis	(ii) Infarction
(ii) Endoscopy	

Infectious	Traumatic/mechanical
(i) Clostridium difficile	(i) Volvulus
(ii) HIV and AIDS	(ii) Malrotation
(iii) Cryptosporidium	(iii) Intussusception
(iv) Cytomegalovirus	(iv) Obstruction/strangulation
(v) Pneumocystis carinii	(v) Blunt abdominal trauma
(vi) Rotavirus	
(vii) Adenovirus	

Inflammatory/Autoimmune	
(i) Crohn's Disease	
(ii) Ulcerative Colitis	

Other	
(i) Graft versus host	
(ii) Pseudo-obstruction	
(iii) Immunosuppression	
(iv) Iatrogenic	

Pulmonary	
(i) Asthma	
(ii) COPD	
(iii) Cystic fibrosis	

**Table 3:** Findings concerning for mesenteric ischemia and infarction within pneumatosis intestinalis.

Clinical Scenario
(i) Low-flow states
(a) CHF
(b) Sepsis
(c) IV pressors
(d) Hypotension
(ii) Arrhythmias

Past medical history
(i) Vascular disease
(ii) Risk factors for vascular disease: CAD, HTN, hyperlipidemia, diabetes, smoking

Physical examination
(i) “Pain out of proportion to exam”
(ii) Peritonitis

Laboratory data
(i) Elevated lactate/acidemia

Radiographic details
(i) Gas within vasculature
(ii) Linear/crescentic gas pattern
(iii) Small bowel gas
